# The impact of adult children rural–urban migration on left-behind parents' health: Evidence from China

**DOI:** 10.3389/fpubh.2022.951124

**Published:** 2022-09-20

**Authors:** Chi Zhang, Kaiyu Lyu, Xiaoyu Cheng, Chongshang Zhang

**Affiliations:** ^1^School of Public Administration, Zhejiang University of Finance and Economics, Hangzhou, China; ^2^Institute of Agricultural Economics and Development, The Chinese Academy of Agricultural Sciences, Beijing, China; ^3^School of Economics, Zhejiang University of Finance and Economics, Hangzhou, China

**Keywords:** adult children migration, left-behind parents, physical health, mental health, remittances, rural China

## Abstract

Population aging and rural–urban migration have posed challenges to the elderly support system in developing countries that lack social safety net and services. Given that there is no consistent conclusion in the literature on whether adult children's internal migration can improve or impair their left-behind parents' health, little is known about the effect mechanisms. This paper investigates the comprehensive impact of adult children's migration on the health of their parents in rural China by analyzing the income effect and time allocation effect. The empirical analysis uses the rural sample of the China Health and Retirement Longitudinal Study (CHARLS) in 2013, 2015, and 2018. We found that adult children migration can improve parents' physical health, mainly thanks to the income effect. The analysis of the mechanism found that although the income effect has a positive effect on parents' health, the time allocation effect has a negative effect on parents' health because of the lack of care and increased working hours of parents. Our findings suggest that providing social care services and enhancing intergenerational communication can be practical strategies to mitigate the negative effect of children's migration to rural left-behind elderly parents.

## Introduction

Population aging and rural–urban migration have posed potential challenges to the elderly support system in developing countries that lack institutional support. As the largest developing country in the world, China has been experiencing rapid population aging, which is more rapid in rural areas than that in urban areas (1, 2). In 2020, China's population of 60 years or older accounted for 18.70% of the total population, and this proportion in rural areas reached 23.81% (2), while elderly care in developing countries much relies on the family, particularly in Asia countries with filial piety culture (3–5), because of the absence of adequate social safety net and service. Along with the aging population, China has also been experiencing rapid urbanization, and massive rural laborers have migrated to cities. In 2020, there were 285.6 million rural-to-urban migrants (6). Out of the total aging population, over 37% are left behind in the rural areas because of the massive migrant labor (7). There is a geographical distance between the migration of adult children and their parents, which makes it difficult for left-behind parents to obtain care and support from their children.

In China, internal migration for a better job is an important way to increase income for many rural households (8, 9). Rural–urban migration is often considered by young individuals as a substitute for acquiring further education (10, 11). There is an obvious trend to provide money rather than companionship and care in supporting the elderly. In rural China, it is often observed that the parents receive more remittances from their migrant children (12–14). Received remittances could improve their nutritional level and improve their health (3, 15). However, migration increased the distance between parents and children, thus making the children less likely to take care of their left-behind parents (16). Due to the absence of children in their life, parents not only need to spend more time on household chores and agriculture production but also increase their loneliness and psychic burden (17). Therefore, it is unclear the exact effect of adult children's migration on their parents' health.

Scholars have increasingly been interested in the influence of adult children's internal migration on their left-behind parents' health, but their conclusions are not consistent. Some literature found that adult children migration can improve the left-behind elderly parents' physical and mental health (18–20). Oppositely, some studies have proved that adult children's migration significantly impairs the health of their parents (3, 16, 21). Other literature has furtherly explored the mechanism by which adult children migration exerts health effects. Böhme et al. found that the transnational migration of adult children could improve their parents' health by significantly reducing agricultural working hours and increasing leisure time (22). Yi et al. found that migrants' remittances can compensate for the adverse health effects of adult children's migration on their parents (4). However, such work remains scarce. In particular, few studies systematically investigate the mechanism of the effect of adult children's migration on their parents' health.

This paper contributes to the literature by empirically demonstrating there are two mechanisms, the income effect and the time allocation effect, of adult children's migration on parents' health. Meanwhile, this paper explores the interaction impact of these two mechanisms on parental health. Although the interaction impact of the two mechanisms is of importance to understand exactly how adult children's migration affects parents' physical and mental health, many existing studies have ignored it. This paper intends to fill the gap. In this study, we investigate the comprehensive impact of adult children migration on the health of their elderly parents in rural China by analyzing the income effect and the time allocation effect, and their interaction.

## Theoretical framework

The aged social security system of rural China has not yet been perfected (7, 19). Rural elderly care relies a lot on adult children (4). Adult children's financial support and care are two essential factors for parents' health (23–25). However, as the distance between migrant children and their left-behind parents increased, the support behaviors of migrant children, which affect the health of their parents, were adjusted accordingly (3).

Adult children's migration may affect their parents' health in two ways. The first way is parents may adjust their time arrangements for agricultural production and domestic labor after the children's migration, which is referred to as the “time allocation effect” (22). As an important labor force in the family, adult children's migration will increase the parents' time required for agricultural production (26). As an important provider of parental support, adult children's migration not only decreases parental care support but also increases the time that parents spend on domestic labor (7, 27). Especially, the Chinese left-behind parents often bear onerous tasks of caring for grandchildren (28–30). Heavy production and domestic labor may cause parents to become unable to take care of their own health and enjoy their leisure time, which has a negative impact on their physical and mental health (31–34). In addition, the lack of migrant children's care and spiritual support aggravates the parents' feelings of loneliness (15). Therefore, the “time allocation effect” produced by adult children's migration may have a negative impact on the left-behind parents' health.

The second way is that parents may receive more remittances from their migrant children, which could have a positive effect on their parents. This effect is referred to as the “income effect” (35). The migration of labor has led to an obvious trend of monetization in the supply of traditional family support. In general, in rural households with migrated labor, the parents could receive higher intergenerational remittances (12–14). As a compensatory family support resource, the remittance by migrant children is not only an important way to maintain the intergenerational relationship but also the most important source of parents' income. As a result, parents can afford to pay higher costs for improving their health, such as diet, leisure, or medical care (3, 15). According to the theory of health economics, more health inputs will improve health (36, 37).

These two ways can influence each other. On the one hand, migrant children's remittance can reduce the parents' labor supply time and, to a certain extent, alleviate the negative impact of “time allocation effect.” The reason is that the employment income of the migrant children can replace or compensate for agricultural income and reduce their family's dependence on agricultural production. Families can reduce the agricultural working time of the elderly parents through land transfer. On the other hand, parents who take care of their grandchildren not only need to input time and energy but also need to bear economic costs, which weakens the positive impact of the “income effect.” Babysitting grandchildren is highly correlated with obtaining financial support from the offspring. Migrant children may increase their financial support to their parents because their parents help to take care of their children (38), and parents use the remittance to cover the daily expenses of caring for their grandchildren, which may weaken the positive impact of the “income effect” on the health.

**Figure 1:**
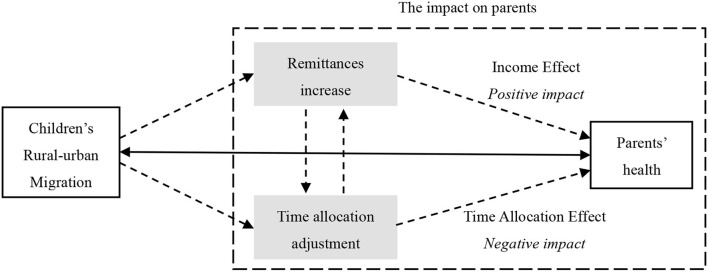
Theoretical mechanism of the impact of children's migration on their parents' health.

Obviously, adult children's migration affects parents' health through two opposing mechanisms. The overall health effect of children's migration depends on the respective strengths of these two mechanisms. That is, if the positive impact of the “income effect” is greater than the negative impact of the “time allocation effect,” children migration will improve the health of their parents. Conversely, adult children migration will worsen the health of their parents. Figure 1 shows the theoretical mechanism of the impact of children's migration on their parents' health. This paper used empirical analysis to evaluate the overall effect of children migration on parents' health and verify the effects of these two mechanisms on parents' health.

## Methods

### Data

The data in this paper are from the China Health and Retirement Longitudinal Study (CHARLS) organized by Peking University. The CHARLS is a nationwide social panel survey project that is specifically aimed at aging individuals in the Chinese population. The survey subjects are middle-aged and elderly people who are 45 years old and older. The project collects data at three levels: the individual, family, and community (village) levels. The CHARLS gathers data on personal health status, medical care, work and retirement, family income, population information, and intergenerational support for children, as well as the community (village) economy and population information. The sample covers 28 provinces in China. Since the 2011 national baseline survey, the CHARLS has been collecting data every 2 or 3 years. This article mainly used the mixed cross-sectional data from the 2013, 2015, and 2018 CHARLS. The response samples in the 2013, 2015, and 2018 CHARLS are 18,264, 20,284, and 19,816, respectively.

People who live in rural areas and have at least one adult child who is 45 years old and older were selected as the research sample. The CHARLS defines the urban and rural samples according to the urban–rural division code of the National Bureau of Statistics. After excluding the urban sample and childless sample, the sample size was 33,460. To obtain village-level information to control the influence of village-level economic and social factors, participants in the sample described above were matched with the data at the community (village) level from the 2011 CHARLS. After matching, there were a total of 30,261 participants.

### Model specification

To evaluate the total effect of adult children migration behavior on their parents' physical and mental health, this paper used the following constructed econometric model:


(1) $$\begin{array}{r} H_{i}\;=\;\beta M_{i}\;+\;\theta Z_{i}\;+\;\varepsilon_{i} \end{array}$$

where *H*_*i*_ is parent *i*'s health level; *M*_*i*_ is an indicator of children's migration situation of parent *i*; *Z*_*i*_ indicates control variables, specifically including the variables of parental characteristics, children and family characteristics, and village-level characteristics, as well as year dummy variables and province dummy variables; and ε_*i*_ is an error term.

The model may have the problem of endogeneity because of the reverse causation between children's migration and the health of their parents. For instance, the poorer health of the parents is, the less likely the children are to work outside the home because they need to stay with their parents to provide care and support. Alternatively, parents with poor health may have a low economic income, which requires their children to migrate for work to earn income. This means that parents' health status affects their children's decision to migrate, which may lead to biased and inconsistent estimation results of the variables for children's migration. The abovementioned endogeneity problem has been fully discussed in existing studies and is usually dealt with by the instrumental variable method (21, 39). Drawing on existing studies, this paper selected the village-level rate of migration and the urban unemployment rate as the instrumental variables of children's migration and performed two-stage least squares estimation (2SLS) (22).

Theoretically, these two instrumental variables meet the conditions of correlation and exogenous. In terms of correlation, in a rural area, the migrant network in the village is an important source of information and social capital for farmers to seek jobs outside of the village. It can not only effectively reduce the cost of employment outside the village but also improve the probability of employment. Generally, the higher the rate of village-level migration is, the greater the possibility of their children's migrating for work. There are two possible effects of the urban unemployment rate on rural employment. The first is that the high unemployment rate in the local city promotes the transfer of rural labor workers to other cities to engage in non-agricultural work; the other is that the high urban unemployment rate causes the labor workers to stay in the countryside to engage in agricultural production. We do not discuss which of the two influences is dominant, but the above analysis shows that the urban unemployment rate is related to rural labor migration. In terms of exogeneity, village-level rate of migration and urban unemployment rate usually do not directly affect an individual's health. In fact, the village-level rate of migration and urban unemployment rate is related to the levels of economic and social development of a region, which may affect an individual's health. Therefore, we controlled for parental income, village-level characteristics, and province dummy variables in the econometric model, which could largely eliminate the endogeneity of instrumental variables.

To evaluate “time allocation effect” and “income effect,” this paper used a multiple-step multiple mediator model. Hayes (2009) stated that when there are multiple mediation variables and the mediation variables interact with each other, a multiple-step multiple mediator model needs to be constructed to test for mediating effects (40). The following multiple-step, multiple mediator model was constructed:


(2) $$\begin{array}{r} C_{i}\;=\;a_{1}M_{i}\;+\;\theta_{1}Z_{i}\;+\;\varepsilon_{1i} \end{array}$$

(3) $$\begin{array}{r} S_{i}\;=\;a_{2}M_{i}\;+\;dC_{i}\;+\;\theta_{2}Z_{i}\;+\;\varepsilon_{2i} \end{array}$$

(4) $$\begin{array}{r} H_{i}\;=\;c^{{}^{\prime }}M_{i}\;+\;{b_{1}C}_{i}\;+\;{b_{2}S}_{i}\;+\;\theta_{3}Z_{i}\;+\;\varepsilon_{3i} \end{array}$$

where *C*_*i*_ is intergenerational care, *S*_*i*_ is children's financial support, and ε_1*i*_, ε_2*i*_, *and ε*_3*i*_ are random disturbance terms. It should be noted that the mediating effect of time allocation adjustments is analyzed in this paper using intergenerational care time as an example. The reasons are as follows: First, in terms of labor time allocation, intergenerational care time is more affected by children's migrating behaviors; second, grandparenting is also caused by the “incompleteness” of labor transfer, and in rural areas, intergenerational care is becoming increasingly common, so it is significant to study the impact of intergenerational care on parents' health (41). Third, when analyzing the mediating effect of labor time allocation, it is necessary to analyze the impact of labor time on health, but there may be an endogenous problem between labor time and health caused by reverse causation. Moreover, it is not beneficial at the technical level to find suitable instrumental variables to address the potential endogeneity, either by summing up the various types of labor time or by analyzing the impact of agricultural labor time on health separately.

Meanwhile, to overcome the potential endogeneity of children's migration behaviors in Equation (2), the same instrumental variables used above were selected, and Equation (2) was estimated using 2SLS. In Equation (3), migration and grandparenting time were also potentially endogenous variables, and the theoretical analysis partially suggested that the financial support provided by migrant children also influences parents' grandparenting behaviors. To this end, drawing on existing research, the number of grandchildren under 16 years of age and the average grandparenting time at the village level excluding the subject himself/herself were selected as the instrumental variables for grandparenting time (28, 29). Equation (3) was estimated using three-stage least squares (3SLS). Parental health status not only affects children's migration behaviors but may also affect children's financial support and parents' intergenerational care behaviors, so there are three potential endogenous variables (migration, children's financial support, and grandparenting time) in Equation (4). Based on the existing studies, the average of children's financial support at the village level excluding the subject himself/herself, and whether they are invited to participate in celebrations and ceremonies are the instrumental variables for children's financial support (16). Equation (4) was estimated using 3SLS.

### Variable specification

#### Health measures

Since health is multidimensional (22), this paper selected multiple variables to measure the parents' physical and mental health. We measured physical health status in three ways: self-reported health (SRH), instrumental activities of daily living (IADL), and physical activity ability. SRH is a comprehensive evaluation indicator of physical health. It has a predictive effect on the individual's future morbidity and mortality (42, 43). However, as a subjective index, the main disadvantage of SRH is that it is easily affected by factors such as individual characteristics and social environments (44). Therefore, we also chose IADL and physical activity ability from an objective perspective to measure physical health. The above two variables were set in the form of the number of parents that could independently complete the activities. The larger the value, the better the health of the parents (19).

We chose the degree of depression and subjective well-being to measure the parents' mental health. The degree of depression was measured by the Depression Scale developed by the Center for Epidemiological Survey (CES-D) (45, 46). The CES-D score ranges from 10 to 40. The higher the score, the higher the degree of depression of the parents and the lower level of mental health. Subjective well-being (SWB) is also an index that has also been commonly used to measure mental health in related studies (16, 47, 48).

#### Migration measures

Migration was the key independent variable. Children's rural–urban migration is often expressed as long-distance geographical migration, that is, children's working and living places are transferred from rural areas to urban areas or to places that are far away from their hometowns. Migration is also manifested in the employment sector, that is, the transfer from the agricultural sector to the non-agricultural sector. Based on the existing literature, migration is defined as having non-agricultural employment with their habitual residence outside of the county where their parents habitually reside. The migration variable was set as a dummy variable (18, 25, 39).

#### Control variables

Considering that there are many factors that affect parents' health, we controlled for three types of independent variables: parental characteristics, children and family characteristics, and village-level characteristics (4, 19–22). Parental characteristics included seven variables: age, sex, years of education, whether spouses lived together, childhood health status, chronic diseases, and whether they had pension insurance. The two variables of childhood health and chronic diseases could reflect the initial health level of an individual and the genetic status of family health to a certain extent. The characteristics of children and family specifically included five variables: the number of children, the average age of the children, highest education level of the children, family income, and whether children were living together. The economic and social development levels of the village where their parents lived could also affect their health. In this paper, four variables were selected to measure village-level characteristics: the village's enterprise, activity facilities, elderly assistance organizations, and whether the residents of the village mainly used flushing toilets. To exclude the influence of the time effect and regional differences, the paper further controlled the year dummy variable and the province dummy variable.

In addition, this paper also examines the effects of the “income effect” and “time allocation effect” on parents' health. Two types of mediation variables were used in the article: One is the remittance provided by the children to their parents, and another is the grandparenting time variable from the perspective of labor supply Table 1 shows the definitions of all the variables.

**Table 1:** Measurement of main variables.

**Variables**	**Description**	**Form/Units**
**Health measures**		
SRH	The interviewee's self-reported health	Very poor = 1, poor = 2, fair = 3, good = 4, excellent = 5
IADL	The number of interviewees who can independently complete 5 activities, including housework, cooking, shopping, making phone calls, and taking medicines	Number
Physical activity ability	The number of interviewees who can independently complete 5 activities, including jogging, walking, climbing stairs, bending the body, and lifting heavy objects	Number
CES-D	The CES-D score of the interviewee	Score
SWB	The subjective well-being of the interviewee	Not at all satisfied = 1, Not very satisfied = 2, Somewhat satisfied = 3, Very satisfied = 4, Completely satisfied = 5
**Independent variables**		
Migration	Whether the interviewee's children have migrated	No = 0, Yes = 1
Age	The age of the interviewee	Years
Sex	The sex of the interviewee	Female = 0, Male = 1
Education	The years of education of the interviewee	Years
Married	Whether the interviewee is married	No = 0, Yes = 1
Childhood health	Interviewee's health condition at 15 years old and younger	Very poor = 1, Poor = 2, Fair = 3, Good = = 4, Excellent = 5
Chronic diseases	The number of chronic diseases the interviewee has	↑
Pension	Whether the interviewee has participated in pension insurance	No = 0, Yes = 1
Social activities	In the past month, did the interviewee engage in the following social activities: socializing with friends, helping relatives and friends, surfing the internet	No = 0, Yes = 1
Number of children	The number of children of the interviewee	Number
Children's age	Average age of the children	Years
Children's education	The highest level of education of the interviewee's children.	Illiteracy = 1, Elementary school = 2, Middle school = 3, High school/Vocational school = 4, College = 5, Postgraduate = 6
Household income	Interviewee's family income in the past year, including agricultural production, non-agricultural production and operation, wages, and transferred income In addition, the logarithmic form of income	Yuan, logarithm
Residence	Whether the interviewee lives with one of their children	No = 0, Yes = 1
Enterprise	The number of enterprises in the residential village	Number
Activity venues	The number of sports, cultural and entertainment venues in the residential village.	Number
Elderly assistance organizations	Whether there are nursing homes, health care centers for elderly individuals, home-based elderly service stations, and other organizations that help the elderly population in the residential village	No = 0, Yes = 1
Toilet type	Whether the main type of toilet in the resident village is a flushing toilet	No = 0, Yes = 1
**Intermediary variables**		
Remittance	The number of financial support measures (including cash and in kind) that the interviewee received from all their children in the past year	Yuan
Grandparenting time	The amount of time interviewee spent caring for their grandchildren in the past year	Hours
**Instrumental variables**		
Village migration proportion	The ratio of the number of migrant people to the total population in the residential village	%
Urban unemployment rate	Urban unemployment rate in the residential city (data from the statistical yearbook of each city or a government work report)	%

## Results

### The effect of children's migration on parents' health

Table 2 shows the descriptive statistical results of the major variables. In general, the mean value of the parents' self-rated health was 2.74, which indicates that the self-rated health of middle-aged and elderly people in rural areas is between “bad” and “fair.” The mean values of IADL and physical activity ability were 4.51 and 4.97, respectively, which indicated that the number of IADL and the physical activities that rural middle-aged and elderly individuals could independently complete was approximately 5. According to the definition of depression, a CES-D score > 20 was considered to indicate depressive symptoms. The mean value of the parents' depression degree was 19.86. Based on calculation, approximately 38% of rural middle-aged and elderly people were suffering from mental depression. The mean value of the parents' life satisfaction was 3.23, which indicates that middle-aged and elderly people in rural areas were generally satisfied with their current life. The results of the group analysis show that there is no significant difference in physical and mental health between the parents with children at home and the parents with children away from home.

**Table 2:** Descriptive statistical analysis of the main variables.

**Variables**	**All samples**			**No migrant children**		**At least one migrant child**		**Difference**
	**Observation**	**Mean**	**S.D**.	**Mean**	**S.D**.	**Mean**	**S.D**.	
**Health measures**								
SRH	29,454	2.74	1.05	2.68	1.05	2.78	1.04	−0.10^***^
IADL	30,254	4.51	1.06	4.51	1.06	4.51	1.06	0.00
Physical activity ability	30,240	4.97	1.87	5.03	1.85	4.92	1.89	0.10^**^
CES–D	30,236	19.86	8.33	19.14	6.98	20.45	9.25	−1.31^***^
SWB	30,230	3.23	0.80	3.22	0.79	3.23	0.80	−0.01^**^
**Independent variables**								
Migration	30,261	0.55	0.50					
Age	30,261	61.08	9.65	60.10	9.87	61.88	9.39	−1.78^***^
Sex	30,261	0.48	0.50	0.48	0.50	0.48	0.50	−0.01
Education level	30,261	4.57	3.98	4.57	3.94	4.58	4.01	−0.01
Married	30,261	0.87	0.33	0.87	0.34	0.88	0.33	−0.01^***^
Childhood health	29,889	6.99	61.00	5.42	46.26	8.27	70.68	−2.85^***^
Chronic disease	30,261	1.14	1.33	1.25	1.38	1.06	1.28	0.19^***^
Pension	30,261	0.83	0.37	0.80	0.40	0.86	0.35	−0.06^***^
Social activities	30,261	0.36	0.48	0.36	0.48	0.36	0.48	0.00
Number of children	30,261	3.07	1.69	2.75	1.45	3.33	1.83	−0.58^***^
Children's ages	30,206	33.85	9.61	32.67	10.48	34.80	8.72	−2.12^***^
Children's education levels	29,657	3.38	1.09	3.25	1.03	3.49	1.13	−0.24^***^
Household income	30,261	7.61	3.41	7.31	3.70	7.85	3.14	−0.54^***^
Residence	30,261	0.48	0.50	0.66	0.47	0.34	0.47	0.33^***^
Enterprises	29,912	2.91	8.27	3.53	9.47	2.40	7.10	
Activity venues	30,261	1.67	2.08	1.83	2.17	1.54	2.01	0.29^***^
Elderly assistance organizations	30,261	0.18	0.38	0.19	0.40	0.17	0.37	0.03^***^
Toilet type	30,261	0.24	0.42	0.25	0.43	0.22	0.42	0.02^***^
**Intermediary variables**								
Remittance	30,261	5244.94	15926.58	4027.93	11642.01	6237.04	18648.53	−2209.11^***^
Grandparenting time	30,261	1523.05	4864.82	1548.36	4895.43	1502.42	4839.77	45.94
**Instrumental variables**								
Village migration proportion	29,850	0.29	0.27	0.26	0.25	0.32	0.27	−0.06^***^
Urban unemployment rate	30,261	3.12	0.75	3.10	0.76	3.14	0.73	−0.03^***^

*** p < 0.01,

** p < 0.05, and

* p < 0.1. We severally adopted Mann–Whitney U–test, chi–square goodness–of–fit test, and two sample t–tests to test the difference for ordinal variables, nominal variables, and interval/ratio variables.

Table 3 shows the estimation results of the effect of adult children's migration on parents' physical and mental health using ordinary least squares (OLS) and 2SLS. The overidentification test showed that we could not reject the null hypothesis that “all instrumental variables were exogenous,” indicating that the instrumental variables in each equation satisfied the exogeneity condition; the F-statistic values of the weak instrumental variable F-test for each equation were all > 10, indicating that each instrumental variable passed the weak instrumental variable test and satisfied the correlation condition. As a result, the instrumental variables were valid.

**Table 3:** Estimated results of the impact of migration on parents' health in rural families.

	**(1)**	**(2)**	**(3)**	**(4)**	**(5)**	**(6)**	**(7)**	**(8)**	**(9)**	**(10)**
	**SRH**		**IADL**		**Physical activity ability**		**CES–D**		**SWB**	
	**OLS**	**2SLS**	**OLS**	**2SLS**	**OLS**	**2SLS**	**OLS**	**2SLS**	**OLS**	**2SLS**
Migration	0.008	0.472^**^	0.036^***^	0.645^***^	0.031	1.850^***^	0.052	0.424	0.011	−0.071
	(0.014)	(0.215)	(0.014)	(0.233)	(0.022)	(0.408)	(0.099)	(1.885)	(0.011)	(0.173)
Age	−0.005^***^	−0.004^**^	−0.022^***^	−0.022^***^	−0.045^***^	−0.044^***^	0.064^***^	0.063^***^	0.005^***^	0.005^***^
	(0.001)	(0.002)	(0.001)	(0.001)	(0.002)	(0.002)	(0.009)	(0.009)	(0.001)	(0.001)
Sex	0.150^***^	0.096^***^	0.169^***^	0.173^***^	0.718^***^	0.723^***^	−2.171^***^	−2.163^***^	0.060^***^	0.059^***^
	(0.013)	(0.017)	(0.013)	(0.014)	(0.022)	(0.024)	(0.102)	(0.103)	(0.010)	(0.010)
Education	0.002	0.010^***^	0.020^***^	0.020^***^	0.046^***^	0.045^***^	−0.169^***^	−0.173^***^	−0.006^***^	−0.006^***^
	(0.002)	(0.002)	(0.002)	(0.002)	(0.003)	(0.003)	(0.014)	(0.014)	(0.001)	(0.001)
Married	−0.026	−0.000	0.032	0.019	0.102^***^	0.066^*^	−1.590^***^	−1.572^***^	0.101^***^	0.102^***^
	(0.019)	(0.025)	(0.022)	(0.023)	(0.034)	(0.038)	(0.174)	(0.178)	(0.016)	(0.017)
Childhood health status	0.000	0.066^***^	−0.000	0.000	−0.000	−0.000	0.002^*^	0.002^*^	−0.000	−0.000
	(0.000)	(0.007)	(0.000)	(0.000)	(0.000)	(0.000)	(0.001)	(0.001)	(0.000)	(0.000)
Chronic diseases	−0.192^***^	−0.184^***^	−0.111^***^	−0.108^***^	−0.328^***^	−0.320^***^	0.816^***^	0.817^***^	−0.061^***^	−0.061^***^
	(0.005)	(0.005)	(0.005)	(0.006)	(0.008)	(0.009)	(0.035)	(0.036)	(0.004)	(0.004)
Pension	−0.016	−0.009	0.068^***^	0.054^***^	0.059^**^	0.015	−0.409^***^	−0.418^***^	0.034^***^	0.037^***^
	(0.016)	(0.021)	(0.016)	(0.018)	(0.027)	(0.032)	(0.122)	(0.134)	(0.013)	(0.014)
Social activities							−0.614^***^	−0.631^***^	0.045^***^	0.045^***^
							(0.093)	(0.093)	(0.010)	(0.010)
Number of children	−0.011^***^	−0.043^***^	−0.018^***^	−0.041^***^	−0.038^***^	−0.107^***^	0.232^***^	0.211^***^	0.007^**^	0.011
	(0.004)	(0.014)	(0.005)	(0.010)	(0.008)	(0.018)	(0.043)	(0.080)	(0.003)	(0.007)
Children's ages	−0.000	0.003^**^	0.002^*^	0.002	0.005^***^	0.005^**^	−0.021^***^	−0.021^***^	0.002^*^	0.001^*^
	(0.001)	(0.001)	(0.001)	(0.001)	(0.002)	(0.002)	(0.008)	(0.008)	(0.001)	(0.001)
**Children's education levels**										
Elementary school	0.023	0.073	0.315^***^	0.234^***^	0.241^**^	0.013	0.408	0.403	−0.110^**^	−0.105^*^
	(0.054)	(0.083)	(0.083)	(0.088)	(0.103)	(0.122)	(0.616)	(0.660)	(0.051)	(0.055)
Middle school	0.046	0.078	0.379^***^	0.275^***^	0.402^***^	0.101	−0.226	−0.240	−0.087^*^	−0.079
	(0.054)	(0.088)	(0.082)	(0.091)	(0.103)	(0.129)	(0.613)	(0.687)	(0.051)	(0.057)
High school/vocational school	0.110^**^	0.129	0.408^***^	0.292^***^	0.541^***^	0.201	−0.655	−0.695	−0.057	−0.048
	(0.055)	(0.093)	(0.083)	(0.093)	(0.104)	(0.135)	(0.618)	(0.712)	(0.051)	(0.060)
College	0.147^***^	0.093	0.424^***^	0.245^**^	0.559^***^	0.035	−1.193^*^	−1.252	−0.031	−0.015
	(0.055)	(0.110)	(0.083)	(0.106)	(0.104)	(0.163)	(0.620)	(0.826)	(0.052)	(0.070)
Postgraduate	0.182^**^	0.050	0.500^***^	0.299^**^	0.682^***^	0.073	−0.542	−0.601	−0.000	0.016
	(0.077)	(0.138)	(0.089)	(0.118)	(0.132)	(0.197)	(0.761)	(0.949)	(0.066)	(0.086)
Household income	0.009^***^	0.008^***^	0.009^***^	0.007^***^	0.023^***^	0.019^***^	−0.100^***^	−0.100^***^	0.010^***^	0.010^***^
	(0.002)	(0.002)	(0.002)	(0.002)	(0.003)	(0.004)	(0.013)	(0.014)	(0.001)	(0.002)
Residence	−0.010	0.095	−0.096^***^	0.072	−0.063^***^	0.437^***^	0.114	0.230	0.019^*^	−0.006
	(0.013)	(0.067)	(0.013)	(0.065)	(0.022)	(0.115)	(0.104)	(0.531)	(0.010)	(0.049)
Enterprises	0.002^**^	0.004^***^	0.001	0.002^**^	0.002	0.005^***^	−0.018^**^	−0.017^**^	0.002^***^	0.002^**^
	(0.001)	(0.001)	(0.001)	(0.001)	(0.001)	(0.002)	(0.007)	(0.008)	(0.001)	(0.001)
Activity venues	0.013^***^	0.016^***^	0.020^***^	0.022^***^	0.021^***^	0.027^***^	0.023	0.019	0.002	0.001
	(0.004)	(0.005)	(0.003)	(0.004)	(0.006)	(0.007)	(0.031)	(0.033)	(0.003)	(0.003)
Elderly assistance organizations	−0.027	−0.055^**^	0.008	0.020	0.027	0.057^*^	−0.242^*^	−0.247^*^	−0.008	−0.014
	(0.017)	(0.023)	(0.017)	(0.018)	(0.029)	(0.033)	(0.136)	(0.141)	(0.013)	(0.014)
Toilet type	−0.013	0.050^*^	0.076^***^	0.108^***^	0.198^***^	0.284^***^	−0.488^***^	−0.491^***^	0.001	0.009
	(0.019)	(0.028)	(0.019)	(0.022)	(0.032)	(0.039)	(0.155)	(0.171)	(0.015)	(0.017)
**Year dummy**										
2015	0.074^***^	0.074^***^	0.021	0.038^**^	0.080^***^	0.126^***^	−0.114	−0.114	0.318^***^	0.317^***^
	(0.015)	(0.016)	(0.015)	(0.016)	(0.024)	(0.029)	(0.087)	(0.098)	(0.011)	(0.012)
2018	0.265^***^		−0.130^***^	−0.316^***^	−0.323^***^	−0.889^***^	3.536^***^	3.431^***^	0.082^***^	0.105^*^
	(0.015)		(0.015)	(0.074)	(0.026)	(0.130)	(0.125)	(0.591)	(0.012)	(0.055)
**Province dummy**	**Controlled**									
Constant	2.733^***^	2.021^***^	5.290^***^	5.108^***^	6.829^***^	6.279^***^	19.639^***^	19.514^***^	2.496^***^	2.533^***^
	(0.082)	(0.149)	(0.104)	(0.130)	(0.146)	(0.203)	(0.791)	(0.996)	(0.072)	(0.091)
Observations	28,330	27,942	28,926	28,533	28,916	28,523	28,912	28,519	28,908	28,516
F value of endogenous test		25.765^***^		7.206^***^		24.454^***^		0.043		0.216
χ2 value of overidentification test		1.407		0.108		0.753		3.604^*^		0.856
F–value of weak instrumental variable test		52.103^***^		52.932^***^		53.118^***^		53.586^***^		53.531^***^

*** p < 0.01,

** p < 0.05, and

* p < 0.1. Robust standard errors are in parentheses; each model controlled for the dummy variables of 24 provinces; illiteracy was used as the reference group for children's education levels.

The empirical results show, in terms of physical health, the estimated coefficient of migration variable was significantly positive in the SRH equation, IADL equation, and physical activity ability equation. The results show that parents' SRH, IADL, and physical activity ability were significantly increased when their children migrated for work. In terms of mental health, the migration variable's coefficient was negative in the CES-D equation and positive in SWB equation. The results show that parents' depression degree was decreased, and subjective well-being was improved when their children migrated for work, but these two coefficients were not statistically significant. This indicates that children's migration does not significantly improve the mental health of their parents.

The estimated results of other control variables were basically consistent with the theoretical and realistic expectations. As the parents grew older, their health gradually declined. There were obvious differences between fathers and mothers. Both the physical and mental health of fathers were significantly better than those of mothers. The higher the status level of childhood health was, the higher the level of physical health and mental health. Chronic diseases have a significant negative impact on parents' health. Income has a significant positive effect on parental health. Furthermore, the more children the parents had, the worse their physical health level was, which indicates, to some extent, that increased child-rearing tasks are not good for parents' physical health. In contrast, with the aging of their children, the health level of parents increased correspondingly. A possible reason is that, after the children have grown, the care burden of parents is reduced on the one hand, and on the other hand, children can also help their parents by sharing labor. From the estimation results of the village-level characteristic variables, it can be seen that village economic and social development levels had a positive impact on the physical and mental health of middle-aged and elderly people.

### Heterogeneous effects of adult children's migration on parents' health

The previous analysis found that children's migration was conducive to improving parents' health. Is there any difference in this effect for parents with different characteristics? Next, we look into the heterogeneous effects on several important dimensions: gender, number of children, and region. Table 4 reports the heterogeneity analysis results, and we focus on the estimates from 2SLS, and we use CHOW test to check the significant difference in the estimated coefficients in different groups.

**Table 4:** Heterogeneity analysis results by 2SLS.

	**(1)**	**(2)**	**(3)**	**(4)**	**(5)**
	**SRH**	**IADL**	**Physical activity ability**	**CES–D**	**SWB**
**Panel A: Gender**					
Father	1.058^***^	0.241	1.257^**^	−3. 172^**^	0.296
Mother	0.469^*^	0.397	0.808	−0.473	−0.008
Chow test	23.49^***^	22.80^***^	16.80^***^	17.39^***^	2.78
**Panel B: Number of children**					
Only one child	−3.811	−1.899	−7.147	7.698	−1.848
More than one child	0.957^***^	0.553^**^	1.456^***^	0.820	−0.121
Chow test	10.06^**^	15.44^***^	9.56^**^	30.13^***^	11.66^**^
**Panel D: Region**					
East	0.068	−0.907	1.483	10.254	−0.960^*^
Middle	1.001^***^	0.969^***^	2.212^***^	−0.514	0.057
West	−3.586^**^	−0.794	−0.779	8.523	−3.391^**^
Chow test	47.13^***^	23.25^***^	18.33^**^	14.78^**^	19.92^***^

*** p < 0.01,

** p < 0.05, and

* p < 0.1. Robust standard errors are in parentheses. Control variables included the three types of variables mentioned above: parental characteristics, children and family characteristics, and village–level characteristics, as well as year dummy variables and province dummy variables.

Panel A shows results separately by male and female. The results based on separated samples show significant differences between father and mother. In the mother group, we can see that most of the absolute values of the estimated coefficients are smaller than those of the father group, and most of the estimated coefficients are insignificant. This indicates that child migration can improve fathers' health, but the improvement in maternal health is not significant. Panel B shows results separately by parents with one child and parents with more than one child. The results show that for parents with one child, children's migration did not have a positive impact on parents' health. But for parents with more than one child, children's migration had a significant positive impact on parents' physical health. Panel C shows results separately by region. According to the standard of the National Bureau of Statistics on the division of administrative regions, the samples are divided into three groups: east, middle, and west. The results of group regression showed that children's migration had a significant positive impact on the health of parents who are living in central rural China and little to no effect on the health of eastern parents. However, it has a significant negative impact on the self-rated health and life satisfaction of parents who are living in western rural China. Overall, our results suggest that children's migration has larger beneficial effects on parents who are male, have more than one child, and live in central rural China.

### The mediation mechanism of adult children's migration on parents' health

Table 5 shows the estimated results of the mediation effect model with all variables normalized. All the instrumental variables used in each equation passed the overidentification test and the weak instrumental variable test, indicating that the instrumental variables were valid.

**Table 5:** Estimated results of the multiple–step, multiple mediator model.

	**(1)**	**(2)**	**(3)**	**(4)**	**(5)**	**(6)**	**(7)**
	**Grandparenting time**	**Remittance**	**SRH**	**IADL**	**Physical activity ability**	**CES–D**	**SWB**
Migration	0.191^*^	0.202^***^	0.468^***^	0.476^***^	−0.001	0.674^***^	1.477^***^
	(0.099)	(0.037)	(0.022)	(0.065)	(0.733)	(0.075)	(0.100)
Grandparenting time		0.903^***^	−0.024^**^	−0.068^*^	−0.241^***^	0.205^***^	−0.371^***^
		(0.123)	(0.010)	(0.041)	(0.048)	(0.045)	(0. 066)
Remittance			0.039^*^	0.630^***^	0.813^***^	−0.883^***^	1.081^***^
			(0.022)	(0.079)	(0.098)	(0.089)	(0.142)
**Independent variables**	**Control**						
Observations	28,540	28,540	27,942	28,533	28,523	28,519	27,935
χ2 value of the overidentification test	0.162	6.924	46.490	10.557	32.672	32.710	157.258
*P*–value of the overidentification test	0.686	0.999	0.973	0.999	0.999	0.895	0.001

*** p < 0.01,

** p < 0.05, and

* p < 0.1. Robust standard errors are in parentheses. Control variables included the three types of variables mentioned above: parental characteristics, children and family characteristics, and village–level characteristics, as well as year dummy variables and province dummy variables.

Columns (1) and (2) in Table 5 show the estimation results of Equations (2) and (3), respectively, that is, the effect of the children's migration on the intermediary variables. The results show that the time of parents' grandparenting time variable and the amount of remittance were significantly increased when their children migrated for work. Also, grandparenting time significantly increased the amount of remittance. The significance of the above variables indicates that intergenerational care and remittance were two effective mediation variables. Columns (3) to (7) in Table 4 are the estimation results of Equation (4), which is the effect of the mediation variable on the outcome variable, that is, parents' health. The results show that the coefficient of grandparenting time variable on CES-D was positive, the coefficients of the other four health measures were all negative, and all were significant at the statistical level of 1%, which indicates that the longer the intergenerational care time was, the higher the degree of depression the parents had, and the lower the SRH, IADL, physical activity ability, and SWB were. This implies that grandparenting time has a significant negative impact on parents' physical and mental health. The coefficient of remittance variable on CES-D was negative, and the estimated coefficients of the other four health indicators were all positive and statistically significant, which indicates that the more financial support children provided, the lower the degree of depression of the parents, and the higher the SRH, IADL, physical activity ability, and SWB were. This means that children's economic support had a significant positive impact on their parents' physical and mental health.

The mediation effects of grandparenting time and remittance were further calculated and compared. The mediation effect of the independent role of grandparenting time could be obtained by calculating the product of a_1 and b_1. Similarly, the mediation effect of the independent role of remittance could be obtained by calculating the product of a_2 and b_2. In addition, grandparenting time also plays a role in the chain-mediated effect by affecting adult children's economic support, which could be obtained by calculating the product of a_1, d, and b_2. The calculated results are shown in Table 6. The results show that the independent mediation effect of grandparenting time on SRH, IADL, physical activity ability, and SWB was negative, and the independent mediating effect on depression was positive. In contrast, the independent mediation effect of remittance on SRH, IADL, physical activity ability, and SWB was positive, the independent mediating effect on the degree of depression was negative, and the absolute value of the independent mediation effect of grandparenting time was smaller than the independent mediation effect of remittance. In addition, grandparenting time also exerted a chain-mediated effect by affecting children's economic support, and the chain-mediated effect and independent mediation effect of grandparenting time were opposite.

**Table 6:** Mediating effect of grandparenting time and children's financial support.

	**SRH**	**IADL**	**Physical activity ability**	**CES–D**	**SWB**
Independent mediation effect of grandparenting time	−0.005	−0.013	−0.046	0.039	−0.071
Independent mediation effect of remittance	0.008	0.127	0.164	−0.178	0.218
Chain mediation effect of grandparenting time	0.007	0.109	0.140	−0.152	0.186

## Discussion

This study examined the question of how adult children's rural–urban migration influences left-behind parents' health using the rural sample of the China Health and Retirement Longitudinal Study (CHARLS) in 2013, 2015, and 2018. This study found that children's migration can improve left-behind parents' physical health, but has no significant impact on parents' mental health, while some previous studies also found adult children's outmigration has positive associations between children's migration and parents' physical health (18, 22), and no significant impact on parents' mental health (49, 50). A possible but not the only reason is that children migration has different effects on health through “income effect” and “time allocation effect.” For the influence of children migration on physical health, the positive effect of children's financial support compensated for the negative effect of time allocation. But in terms of mental health, the positive effect of children's financial support on their parents' mental health cannot completely replace or compensate for the negative effect of the weakened care and mental support caused by the absence of the children on their parents' mental health.

The mediation impact mechanism of children's migration on parents' health is analyzed. The study demonstrated time allocation and children's financial support mediated the relationship between children migration and parents' physical and mental health in opposite directions. The empirical analysis showed that children rural–urban migration negatively affected parents' health by increasing the burden of intergenerational care but positively affected parents' health by increasing financial support, and the positive effect of children's financial support was greater than the negative effect of intergenerational care. In addition, intergenerational care also exerted a positive chain mediating effect by affecting children's financial support; that is, children's financial support could mitigate the negative effects of intergenerational care on parents' health.

This study also found that there were obvious heterogeneous effects on gender, the number of children, and region. Children's migration improved fathers' health but did not improve mothers' health. There are two possible reasons: One is female health is more fragile and easily affected by changes in the external environment; the other is the female has an advantage in the role of caregiver, and mothers are more likely to bear family labor and the task of caring for grandchildren when their children go out to work. Therefore, compared to fathers, mothers' health is more severely negatively affected. We found that there is an adverse effect on parents who are living in western rural China. The possible reason is that in the western regions of China, the labor usually migrates to the eastern coastal areas to work, and the family income may improve after the children migrate. However, due to the distance, the frequency of children returning to their hometown is generally relatively low. Parents lack care and spiritual support and need to undertake more family labor. The income effect of migration cannot compensate for the time allocation effect. Meanwhile, in western rural China, public resources, such as medical and elderly support, are relatively insufficient. Parents need more support from their children.

## Conclusion

In this study, we found that adult children migration can improve parents' physical health, mainly thanks to the income effect. We also found although the income effect has a positive effect on parents' health, the time allocation effect has a negative effect on parents' health because of the lack of care and increased working hours of parents. Meanwhile, the positive income effect can partly offset such negative effects in health caused by the absence of migrant children.

The findings of this study have important implications. First, it is of great importance to promote the free movement of migrant workers. Rural labor mobility can optimize resource allocation, transfer surplus labor, significantly increase farmers' income, and subsequently, has a positive effect on the physical health of the rural elderly population through the income effect. Therefore, the policy needs to be improved to promote rural surplus labor transfer, including enhancing labor training, rights and interest protection, and welfare and household registration system reform. Second, intergenerational communication and relevant social care services for the rural left-behind elderly population should be enhanced furtherly. Although the transfer payment from migrant children can mitigate the negative effect of children's migration, it is quite limited to parents' mental health. Currently, the mental health of the rural left-behind elderly population in China is easily overlooked, this population's mental health problems are more serious. Giving financial support cannot completely substitute for the absence of children, and the communication between migrate children and left-behind parents should be enhanced with the application of modern information technology. Moreover, the social care system also should be constructed to provide excellent care service for the left-behind parents in rural areas.

## Data availability statement

The original contributions presented in the study are included in the article/supplementary material, further inquiries can be directed to the corresponding author.

## Author contributions

ChiZ designed the study, carried out the data collection and analysis, and participated in drafting the manuscript. ChoZ was involved in data analysis and drafting the manuscript. KL and XC participated in drafting the manuscript. All authors contributed to reviewing the manuscript and have read and agreed to the published version of the manuscript.

## Funding

This research was funded by the Soft Science General Program of Zhejiang Province, China (Grant Number: 2022C35096) and the National Natural Science Foundation of China (Grant Number: 72003186).

## Conflict of interest

The authors declare that the research was conducted in the absence of any commercial or financial relationships that could be construed as a potential conflict of interest.

## Publisher's note

All claims expressed in this article are solely those of the authors and do not necessarily represent those of their affiliated organizations, or those of the publisher, the editors and the reviewers. Any product that may be evaluated in this article, or claim that may be made by its manufacturer, is not guaranteed or endorsed by the publisher.
